# Multimodal Imaging of Acute Central Retinal Artery Occlusion

**Published:** 2019-10-01

**Authors:** Marwa Mahmoud Abdellah

**Affiliations:** 1 Department of Ophthalmology, Faculty of Medicine, Sohag University, Sohag, Egypt

**Keywords:** Optical Coherence Tomography, OCT, Retinal Infarction, Retinal Ischemia, Central Retinal Artery Occlusion

## Abstract

The aim of this study was to describe fluorescein angiography (FA), ocular coherence tomography (OCT) and ocular coherence tomography angiography (OCTA) in the diagnosis of acute central retinal artery occlusion (CRAO). This is an observational case series study performed at Sohag Ophthalmic Investigation Center. Fifteen patients presented by a sudden marked unilateral diminution of vision were included. Corrected Distance Visual acuity (CDVA), color fundus photos, FA, OCT and OCTA, imaging obtained in the first week of presentation and imaging of the other normal eye as a control were assessed. Central macular thickness (CMT), parafoveal inner retinal layers thickness and parafoveal outer retinal thickness in diseased and contralateral normal eyes were compared. Fifteen patients (mean age 52.67 years, 11-74 years old) including 66.7% male entered the study. CDVA ranged from no perception of light to 0.05 (20/400). Fundus examination showed a cherry red spot in 10 cases (66.7 %) and retinal whitening in 9 cases (60%), arteriolar narrowing in 7 (46.67%), optic disc edema in 4 (26.67%), optic disc pallor in 5 (33.3%) and cattle trucking in 5 (33.3%). Fluorescein angiography showed delayed arteriovenous transit time > 23 seconds in 8 cases (53.33 %) and normal FA in 4 cases (26.67 %). OCT revealed increased hyperreflective of the inner retinal layers in comparison to hyporeflective inner retinal layers in all cases (100%) and significant increase in CMT in 10 cases (66.67%). The mean ± standard deviation (SD) of CMT (CRAO) was 306.5 ± 27.9 (P < 0.001), the parafoveal inner retinal thickness (CRAO) 345 ± 51.8 µm (P < 0.001) and the parafoveal outer retinal thickness (CRAO) 120.9 ± 13.6 µm (P < 0.001). OCTA was performed and clear images obtained in 11 cases (73.33%). Disruption of superficial and deep capillary plexus was found in all cases. We concluded that the OCT is the most confirmative imaging method in the diagnosis of acute CRAO even in the absence of fundus signs. OCTA confirms the diagnosis, but it cannot be performed in some cases.

## INTRODUCTION

One of the most important ophthalmic emergencies manifesting by as sudden unilateral vision loss is retinal artery occlusion. Retinal artery occlusion is a common eye disease. It is classified according to involved anatomical site and underlying pathology to central retinal artery occlusion (CRAO), branch retinal artery occlusion and ciliary retinal artery occlusion. CRAO is the most commonly occurring type of retinal artery occlusion. It is important because it causes a permanent sudden acute and painless vision loss. The main etiologies of CRAO are embolus, inflammation and arteriosclerosis [1-3]. Central retinal artery occlusion pathologically is classified to non-arteritic permanent CRAO which forms the majority of cases and non-arteritic transient CRAO which is known as amaurosis fugax and represents 15% of cases and carries the best prognosis [4]. The third most prevalent one is non-arteritic CRAO with cilioretinal sparing which affects about 30% of eyes presented by CRAO who have a patent cilioretinal artery with nourished central macula and papillomacular bundle. The last category is arteritic CRAO associated with vasculitis [5]. Acute CRAO is usually diagnosed based on history and clinical ﬁndings. However, some cases of acute retinal artery occlusion may be associated with a normal-looking fundus which is a diagnostic challenge [6]. An early definitive diagnosis of the CRAO is needed, to allow early measures to reestablish circulation and restore vision if possible. Fluorescein angiography (FA) was considered the cornerstone for studying retinal vascular circulation and ischemia evaluation but it cannot evaluate deep ischemia or identify insults to deep capillary plexus [7]. However, FA was studied recently and patients with CRAO are classified to “poor perfusion >23 seconds transit time, “exudative type” with normal circulation and mixed type [8]. Optical coherence tomography (OCT) is a non-invasive, non-contact imaging technology used to study retinal layers’ anatomy. Many signs have been observed and recorded by OCT examination in patients with CRAO [9]. However, OCT-angiography (OCT-A) is a recent technology used to study vascular maps of the retina which allows a good evaluation of superficial and deep capillary plexuses [10]. The aim of our study was to describe different imaging modalities in the diagnosis of acute CRAO.

## METHODS

Fifteen patients presented by a sudden onset unilateral diminution of vision presented to Sohag Ophthalmic Investigation Center, Egypy from January 2018 to June 2018 were included in this study. The time elapsed from initiation of symptoms to examination ranged from few hours to 7 days with an average of 2.33 days. Exclusion criteria were any concurrent retinal pathology in the diseased eye or contralateral eye as diabetic retinopathy or age-related macular degenerations. Diagnosis of acute CRAO depended on the history of sudden monocular vision loss, retinal whitening and cherry red spot in fundus examination, delayed arterial filling on FA and hyper-reflectivity of the inner retinal layers on OCT [11]. Corrected Distance Visual acuity (CDVA) was measured using decimal values, anterior segment examination performed using slit lamp and color fundus photo performed by a TOPCON retinal camera (TRC-NW400 Non-Mydriatic Retinal Camera Oakland, NJ, USA). Fluorescein angiography (FA) examination was performed as follows; Tropicamide was administered for mydriasis. FA examination was performed using Topcon Imagenet Fundus Camera TRC-NW400 Non-Mydriatic Retinal Camera Oakland, NJ, USA). Sodium fluorescein 20% administered by intravenous infusion. The arteriovenous phase time was calculated as the difference between the start of central artery perfusion and appearance of retinal vein laminar flow.

 Ocular coherence tomography (OCT) of the macula and OCT angiography (OCTA) performed using the AngioVue XR Avanti system (Optovue Inc., Fremont, CA, USA). Central macular thickness (CMT) was measured. The parafoveal inner and outer retinal thickness were measured at 1000 micrometer (µm) from the foveola and the results between diseased eyes with acute CRAO and contralateral normal eye were compared. In OCTA retinal capillary bed was segmented into superficial and deep capillary plexus. Each blood flow angiography image was associated with an en face optical coherence**,** the superficial retinal capillary plexus, which was defined as the region between the vitreoretinal interface and the outer border of the ganglion cell layer; however, the deep retinal capillary plexus, which was found in the area bordered between the inner border of the inner plexiform layer (IPL) and the outer border of the outer plexiform layer (OPL). The deep retinal layer surrounding the fovea shows a spider-web pattern of vessels with many small discontinuous segments [7].

The present study adhered to the tenets of the Declaration of Helsinki and was approved by the ethical committee of Faculty of Medicine, Sohag University. An informed consent was obtained from all patients or their guardians.

Data was analyzed using SPSS version 22.0. (SPSS Inc., Chicago, Illinois, USA). Quantitative data was expressed as means ± standard deviation (SD). Qualitative data was expressed as number and percentage. The data was tested for normality using the Shapiro-Wilk test. Independent Samples t-test was used for normally distributed data. P-value below 0.05 was considered as statistically significant.

## RESULTS

**Figure 1:**
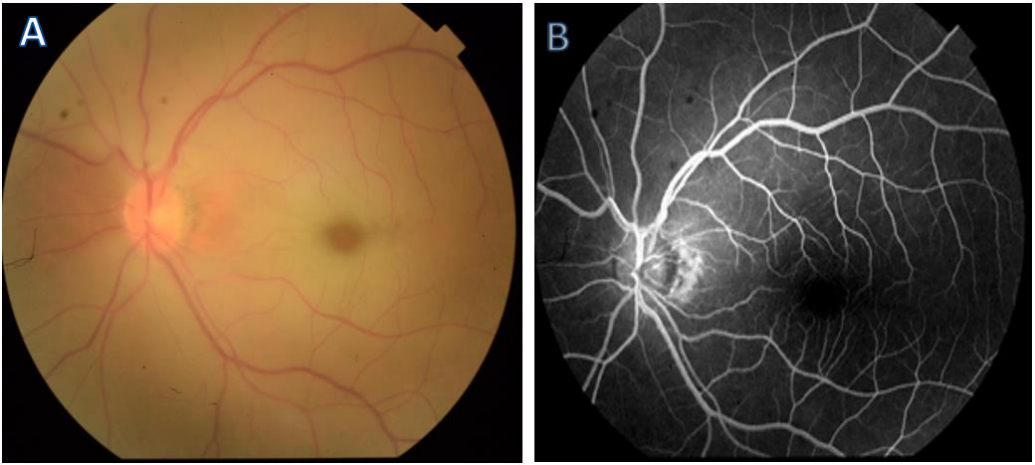
A: shows Color Fundus Photo of the Left Eye (OS) of a 47-Year-old Man presented by an Acute Central Retinal Artery Occlusion 2 Days after Symptom Appear. B: Fluorescein Angiography of the same Patient shows early Fluorescein Angiography with Complete filling of Central Retinal Artery at 24 Seconds

Fifteen patients (mean age=52.67, age range 11–74 years) were included; 66.7% were male. Involved eyes in patients were six in the right (OD) and nine in the left (OS). CDVA ranged from no perception of light (NPL) to 0.05 (20/400). Fundus examination and FA findings are shown in Table 1, Figures 1, 4 and 7.

**Table 1:** Fundus Examination and Fluorescein Angiography Findings

Sign	No. (%)
Cherry red spot	10 (66.7)
Retinal whitening	9 (60)
Arteriolar narrowing	7 (46.67)
Optic disc edema	4 (26.67)
Optic disc pallor	5 (33.33)
Cattle trucking	5(33.33)
Delay of arteriovenous transit time >23 seconds	9 (60%)
Bad peripheral perfusion and peripheral retinal ischemia	8 (53.33)
Optic disc exhibited hyper- fluorescence	9 (60)
Normal appearing fundus	4 (26.67)

No.: number; %: percentage

**Table 2:** The OCT Findings in Acute Central Retinal Artery Occlusion

OCT findings	No. (%)
Increased Central Macular Thickness	10 (66.67)
Loss of Organized Layer Structure	11 (73.33)
Increased Hyperreflectivity of the Inner Retinal Layers	15 (100)
Hyporeflective Outer Retinal Layers	15 (100)

OCT: Ocular Coherence Tomography; No.: number; %: percentage.

**Figure 2:**
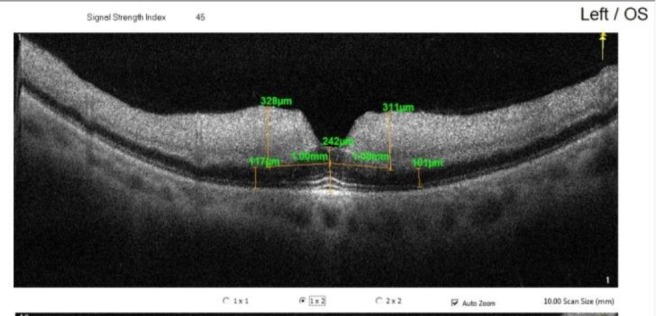
Shows disorganized Hyperreflective Inner Retinal Layers with increased Parafoveal Thickness and Hyporeflective Outer Retinal Layers

OCT showed significant increase in CMT in 10 cases (66.67%). The mean ± SD of CMT was 306.5 ± 27.9 µm compared to 255.2 ± 13.3 µm in contralateral normal eye (P value < 0.001) and increased hyperreflectivity in the inner retinal layers in 100% of cases, with hyporeflective outer retinal layers in 100%. The thicknesses were markedly increased in the inner layers including the retinal nerve fiber layer (RNFL), ganglion cell layer (GCL), IPL and inner nuclear layer (INL) shown in Tables 2 and 3, Figures 2, 5 and 7C.

**Table 3:** Difference of Macular Thickness Measurements between Eyes with Acute CRAO and Contralateral Normal Eyes

OCT findings	Eyes with acute CRAO	Contralateral normal eye	p-Value
CMT	306.5 ± 27.9	255.2 ± 13.3	**< 0.001**
Parafoveal Inner Retinal Thickness in Macula (at 1000µm From the Foveola)	345 ± 51.8	198.3 ± 9.5	**< 0.001**
Parafoveal Outer Retinal Thickness in Macula (at 1000µm From the Foveola)	120.9 ± 13.6	90.4 ± 8.7	**< 0.001**

Data are presented as Mean ± SD.

OCT: Ocular Coherence Tomography; CRAO: Central Retinal Artery Occlusion; CMT: Central Macular Thickness; SD: standard deviation; µm: micrometer; p-value < 0.05 is in bold (independent sample t –test).

**Figure 3:**
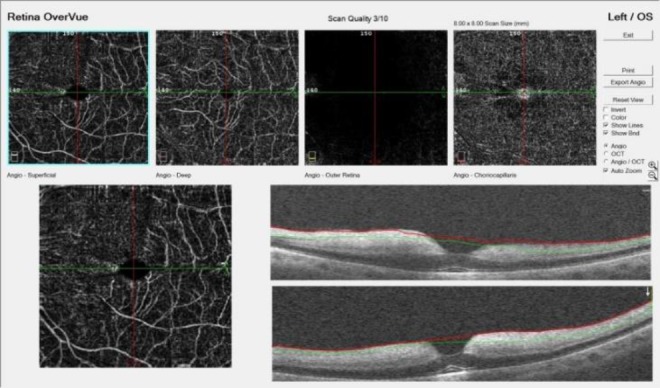
Ocular Coherence Tomography Angiography (OCTA) (8×8 scan size) shows Disruption of the Superficial Retinal Plexus more in the Nasal Side of the Macula, less Disruption in Deep Retinal Plexus, Hyperfluorescence at the Fovea surrounded by Ischemia in the Choriocapillaris Slap

OCTA was performed in all cases and clear images obtained in 11 cases only due to difficult fixation, long acquisition time and motion artifacts. The disruption of the superficial and deep capillary plexus was noted in all cases with decreased vascular perfusion. The superficial retinal plexus was more compromised (Figures 3 and 6).

**Figure 4:**
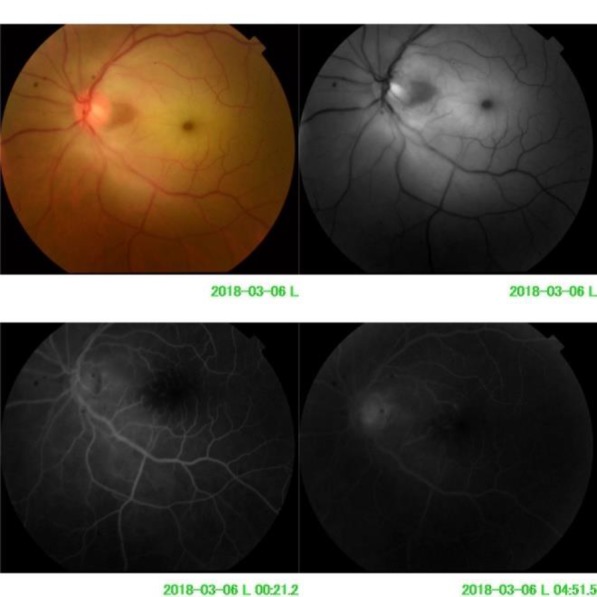
Shows Color Fundus Photo and Fluorescein Angiography of a 53-year-old Female presented by Acute Central Retinal Artery Occlusion 4 Days after Initiation of Symptoms. Color fundus photo of left eye shows Cherry Red Spot, White posterior Pole and Cattle tracking. FA shows Complete filling at 21 Seconds with Optic Disc Hyperfluorescence at Late Stage

**Figure 5:**
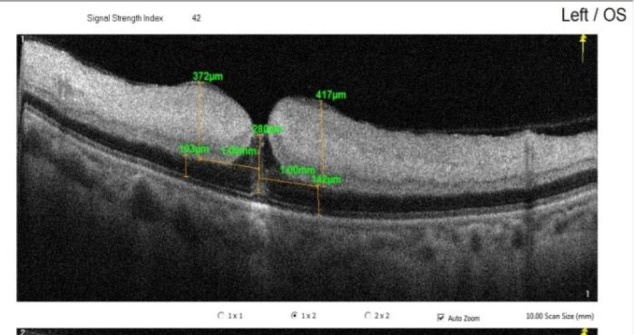
Ocular Coherence Tomography (OCT) Image of the Macula of the same Patient in Figure 4 shows disorganized Hyperreflective inner Retinal Layers with increased Parafoveal Thickness and Hyporeflective outer Retinal Layers

**Figure 6:**
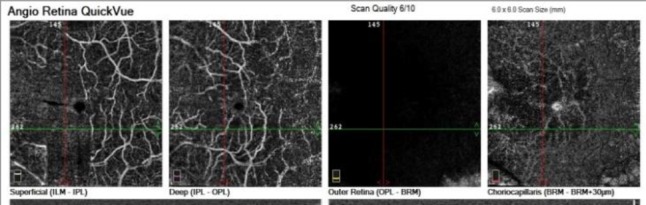
Ocular Coherence Tomography Angiography (OCTA) (6×6 scan size) of the same Female Patient in Figure 4 shows Disruption of the Superficial Retinal Plexus more in the Nasal Side of the Macula, less Disruption in Deep Retinal Plexus, Hyperfluorescence at the Fovea surrounded by Ischemia in the Choriocapillaris Slap

**Figure 7:**
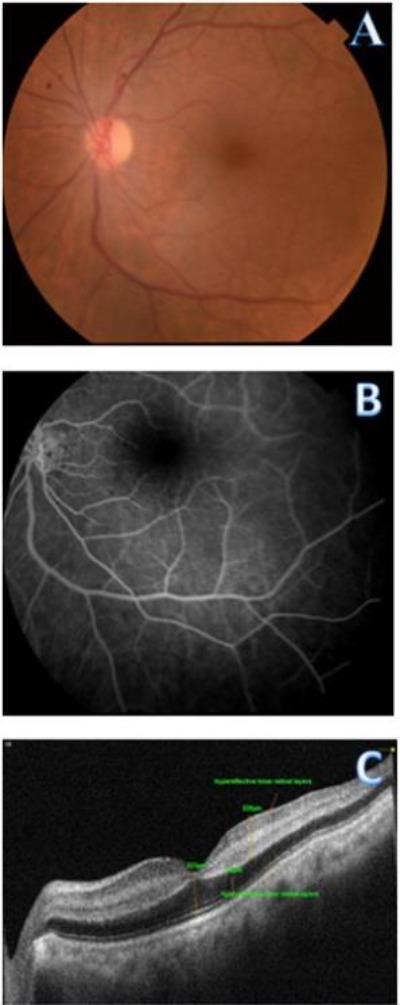
A 43-year-old Male Patient presented by Sudden Onset Unilateral Diminution of Vision in the Left Eye with A: Normal Apparent Fundus, B: Normal Fluorescein Angiography (FA) with Normal Arteriovenous Filling. C: Ocular Coherence Tomography (OCT) shows Hyperreflective Inner Retinal Layers and Hyporeflective Outer Retinal Layers correlates with Acute Central Retinal Artery Occlusion occurred with Relief of the Obstruction, Structural Damage to the Retinal Layers occurred which is Evident by OCT Figure 7C

## DISCUSSION

Fifteen patients with CRAO were enrolled in this study. Fundus examination and FA were variable. The cherry red spots presented in 66.67% of cases, retinal whitening in 60% and optic disc hyper-fluorescence in 60%, poor perfusion in 60%, while 26.67% manifested as normal looking fundus. OCT helped a lot to give more definitive diagnosis by its characteristic picture of increased inner retinal layers reflectivity in all cases with increased CMT and parafoveal thickness in two thirds of cases. OCTA showed disruption in the superficial and deep capillary plexuses at the macula. 

Von Graefes was the first to describe CRAO in 1859 [12]. The central retinal artery (CRA) is described anatomically as a branch of the ophthalmic artery, which is divided into four different subclasses [12]. The incidence of CRAO is reported as 1 in 100 000 people and actually for 1 in 10000 who seeks ophthalmological medical advice as mentioned by Rumelt [13]. CRAO leads to clinically detectable changes of the retina, as the inner retinal layers mainly depend on CRAO in nutrition. Suspicion of the CRAO depends on history and clinical findings observed in fundoscopic examination as CRAO usually presents with sudden, painless monocular sight loss. CDVA equals or less than counting fingers is found in 74% of patients with a visual field defect [13]. However, the fundus photo may be normal in many cases making the diagnosis difficult [6, 14]. So many modalities of investigations including FA, OCT and OCTA have tried to give an early and conclusive diagnosis of CRAO which may contribute in the early management of CRAO.

Fundoscopic findings in CRAO vary based on the time from the event and by the type of CRAO. So in this study, the findings were reported in the first week of initial presentation [15] with an average of 2.33 days and the obtained results seem to be similar to the study performed by Hayreh, but he reported cherry-red spot in 90% while we reported cherry red spots in 66.6% of cases [5, 15]. However, FA showed delayed arteriovenous phase > 23 seconds in 60%, the optic disc exhibited high fluorescence with blurred boundaries in 66.6%, and bad peripheral perfusion and peripheral retinal ischemia in 60%. There were 4 cases of normal fundoscopic picture and normal FA while the CDVA and OCT picture are identical to CRAO which is mentioned in other reports before [14]. It was explained by that the cause of CRAO was emboli, which were quickly degraded and transported to the periphery giving the picture of patent central retinal artery [14] or relieved vasoconstriction occurred in the CRA. But in experimental animal models ischemia has been noted to occur at 240 minutes which led to irreversible ischemia and damage to the GCL [16]. The variation of clinical picture and FA findings can be explained by sub-classification of CRAO which was previously described by Schmidt and his colleagues. They classified CRAO into incomplete CRAO (reperfused manifested by decreased visual acuity, mild retinal edema and thickening and delayed but slightly interrupted blood flow on FA), subtotal CRAO (partially diminished visual acuity, mild retinal edema and thickening and distinct delay in arteriolar blood flow on FA) and total CRAO which presented by no light perception, massive retinal edema and occluded arterioles [5, 17, 18]. Also, Gong et al. classified the perfusion status of FA into poor perfusion, exudative type and mixed type giving a high variation in the FA appearance of CRAO [8]. 

Optical coherence tomography studied retinal architecture in CRAO and evaluated the CMT and parafoveal macular thickness and the architecture of the inner and outer retinal layers [19-21]. In this study, the CMT increased in 66.67% of cases which was significant compare to the contralateral normal eye indicates macular swelling on ischemia, agreed with a previous study by Chen et al. [22] who found CMT increased to 299 ± 76 μm in acute CRAO which was statistically significantly different from the normal contralateral eye. Previously, Yanoff described the pathology of CRAO as an initial ischemic necrosis with intracellular edema at the acute stage and later resulting in the formation of a cellular scar of the inner retinal cell layers [23]. The same has been described by Chen as intracellular edema, rather than extracellular ﬂuid accumulation, accounts for macular edema with clinical grey retinal opaciﬁcation at the acute stage [22]. While the fovea is devoid of ganglion cells as the main structure that explicates the intracellular edema. As the ganglion is highly sensitive to ischemia and clustered around the foveal margin but the central foveal thickness is actually increased which was postulated by Chen who reported the same findings. This occurs due to sequential displacement of neuronal cells toward the fovea and thus deranges the foveal contour and increases the CMT [22]. He also supposed that the size of cherry red spot was variable in the fundus and depends on how much ganglion cells are displaced to the center. 

 In this study, the early OCT examination revealed derangement of the macular contour with increased reﬂectivity of the inner retinal layers on the diseased eyes in comparison to the contralateral normal eye. The optical signals of the outer retina and retinal pigment epithelium/choriocapillaris complex were shadowed by the inner retinal layers which was clearly evident in 100% of cases and totally in agreement with a recent study performed by Feucht who found the inner retinal layers hyperreflectivity in all cases [24]. Higher reflectivity of the inner retinal layers in cases of acute ischemia results in less reflectivity in the outer retinal layers. The exact mechanism of changes in the optical intensity of retinal layers in acute CRAO is still unknown [11]; however, other supposed that the hyperreflectivity in the inner retinal layers may represent activated microglial tissue induced by hypoxia with upregulation of inflammatory molecules in an attempt to repair ischemic tissue at ischemia repair [24]. In the present study we noticed that the greatest optical hyperreflectivity correlates with more ischemia noticed in FA and poor perfusion. The same concept has been previously reported in a recent study [11]. Loss of organized layer structure was found in 60% of cases, mainly in those with bad perfusion and extensive retinal ischemia in FA. This agreed with the OCT finding reported before in other studies [21]. Furashova and Matthé also reported an increased CMT in patients with acute CRAO and classified CRAO into incomplete, subtotal and total types. They linked highly increase in the CMT with total type and reported increased hyperreflectivity of inner retinal layers and decreased reflectivity of outer retinal layers in all cases regardless of the type [11]. However, Ahn et al. [9] reported quantitative data for the diagnosis to avoid physician subjectivity, depended on CMT and parafoveal MT of inner and outer retinal layers. Actually, we did not classify CRAO into grades due to small number of cases and this grading system has been shown to be related to the prognosis.

Loss of organized retinal structure was found in 73.33% of these cases. Furashova mentioned that distinct loss of organized layered structure of the inner retina occurred in total and subtotal types; however, in incomplete CRAO the organized retinal layer structure is preserved with less macular edema [14]. Ocular coherence tomography angiography imaging was performed in all cases, but 11 cases only obtained good OCTA examination. Four cases were lost due to improper images acquisition which mainly related to difficult fixation and long acquisition time to detect blood flow. These obstacles were mentioned before regarding OCTA examination in acute CRAO [5, 25]. OCTA showed marked disruption of superficial capillary plexus and deep capillary plexus with decreased vascular perfusion. We noticed that decreased vascular perfusion was more evident in superficial capillary plexus which is consistent with Boninie study who reported restoration of deep capillary plexus flow in a patient with chronic CRAO with cilioretinal sparing [25]. Other few cases reported OCTA in acute CRAO [26-29] which mentioned disruption in superficial and deep retinal plexuses. Leng et al. reported a patient in whom the diagnosis of CRAO was obtained via OCTA alone [29]. Actually many limitations oppose the use of OCTA in the diagnosis of CRAO. These limitations include longer image acquisition time relative to the standard OCT, poor patient fixation susceptible to motion artifact, lack of normative reference standards data to compare and the structural and optical reflectivity changes occurring during different stages of CRAOs make accurate segmentation of the vascular retinal layers difficult [25]. Other studies suggested the use of OCTA in monitoring and evaluation treatment of acute CRAO [27, 29, 30] to detect restoration of blood flow after an early successful management. 

 Finally, FA is an invasive method with some complications such as nausea, vomiting, dyspnea, syncope and anaphylaxis [31], and may show normal arteriovenous filling with no evident signs. OCT images give more definitive diagnosis with a characteristic picture pathognomonic for acute CRAO or acute macular ischemia, otherwise OCTA interpretation may give a help with the OCT images, but loss of patient fixation with long acquisition time with difficult segmentation give some obstacles which need more investigations. Although, using multiple imaging modalities could be considered as a strength of the current study. However, a small number of the study subjects, lack of long-time follow-up examinations data, and subsequently, lack of statistical values for correlations between used imaging modalities could be some of its limitations. Therefore, we would suggest designing of study focused on this devastating ocular entity with more sample size, longer follow-up time and evaluation of correlation coefficient values between different imaging modalities.

## CONCLUSIONS

Multimodal imaging in CRAO gives an accurate diagnosis. OCT is considered the most confirmative imaging method in the diagnosis of acute CRAO even in the absence of fundus signs. Besides, OCTA confirms the diagnosis, but it cannot be performed in some cases. 

## DISCLOSURE

Ethical issues have been completely observed by the authors. All named authors meet the International Committee of Medical Journal Editors (ICMJE) criteria for authorship of this manuscript, take responsibility for the integrity of the work as a whole, and have given final approval for the version to be published. No conflict of interest has been presented.

## Funding/Support

None.
